# Extended spectrum beta-lactamase producing *Enterobacterales* faecal carriage in a medical intensive care unit: low rates of cross-transmission and infection

**DOI:** 10.1186/s13756-019-0572-9

**Published:** 2019-07-10

**Authors:** Renaud Prevel, Alexandre Boyer, Fatima M’Zali, Thibaut Cockenpot, Agnes Lasheras, Véronique Dubois, Didier Gruson

**Affiliations:** 1 Medical Intensive Care Unit, CHU Bordeaux, Pellegrin universitary hospital, Place Amélie Raba-Léon, F-33000 Bordeaux, France; 2 UMR 5234 CNRS, Bordeaux University, F-33000 Bordeaux, France; 3 Bacteriology laboratory, CHU Bordeaux, F-33000 Bordeaux, France; 4 Hygiene unit, CHU Bordeaux, F-33000 Bordeaux, France

**Keywords:** Extended-spectrum beta-lactamase, Carriage, Cross-transmission, Infection, Ventilator-associated pneumonia, Intensive care

## Abstract

**Background:**

Extended-spectrum beta-lactamases-producing *Enterobacterales* (ESBL-E) are disseminating worldwide especially in Intensive Care Units (ICUs) and are responsible for increased health costs and mortality. The aims of this work were to study ESBL-E dissemination in ICU and to assess the impact of ESBL-E fecal carriage on subsequent infections during a non-outbreak situation.

**Methods:**

We therefore screened every patient at admission then once a week in a medical ICU between January and June 2015. Each ESBL-E isolate was characterized by ESBL genes PCR amplification and the clonal dissemination was assessed by Pulsed-Field Gel Electrophoresis (PFGE).

**Results:**

Among the 608 screened patients, 55 (9%) were colonized by ESBL-E. Forty-four isolates were available for further analysis. Most of them (43/44, 98%) contained a ESBL gene from the CTX-M group. Only one case of ESBL-E cross-transmission occurred, even for acquired ESBL-E colonization. Subsequent infection by ESBL-E occurred in 6/55 (11%) patients and infecting ESBL-E strains were the colonizing ones. ESBL-E faecal carriage had a negative predictive value of 100% and a positive predictive value of 40% to predict ESBL-E ventilator associated-pneumonia (VAP). Alternatives to carbapenems consisting in piperacillin-tazobactam, ceftolozane-tazobactam and ceftazidime-avibactam were all active on this panel of ESBL-E.

**Conclusions:**

ESBL-E expansion and acquisition in ICU in a non-outbreak situation are not any more fully explained by cross-transmission. Mechanisms underlying ESBL-E dissemination in ICU are still to investigate. Interestingly, as far as we know, our study demonstrates for the first time by PFGE that the colonizing strain is indeed the infecting one in case of subsequent ESBL-E infection. Nevertheless, subsequent ESBL-E infection remains a rare event conferring poor positive predictive value for ESBL-E colonization to predict ESBL-E VAP. Relevance of systematic ESBL-E faecal screening at ICU admission and during ICU stay needs further investigation.

## Background

The increase in antimicrobial resistance remains a major threat [1]. Among resistant bacteria, extended-spectrum β-lactamase-producing *Enterobacterales* (ESBL-E) are of special concern. In fact, ESBL-E faecal carriage is increasing worldwide [2, 3] , especially in long term care facilities and ICUs, but even among healthy people with up to ten-fold increase in a 5 years French Survey [4, 5]. ESBL-E faecal carriage rates vary between western and non-western countries probably because of discrepancies in water sanitation procedures (from 1 to 6% in Europe and North America but up to 60% in India) [3] . Known risk factors are previous antibiotic exposure, previous admission to a healthcare facility, previous hospitalization and previous ESBL-E carriage [6–8]. Cross-transmission in ICU has been mainly described during outbreaks leading to enforcement of hygiene isolation procedures [9]. However, hygiene procedures were not able to fully prevent ESBL-E increase in ICU despite increased standard precautions and an efficient prevention from nosocomial cross-transmission [10, 11]. The environment of healthcare facilities (such as floors and walls contamination) was also suspected to play a role in ESBL-E cross-transmission but its role does not seem to be that important [12]. Finally, little is known about the mechanisms of ESBL-E faecal carriage dissemination during ICU stay in a non-outbreak situation when cross-transmission by healthcare workers is controlled by thorough hygiene procedures.

Moreover, to date, the link between ESBL-E faecal carriage and the risk of subsequent ESBL-E infection is not fully understood even if colonization by ESBL *Klebsiella pneumoniae* seems to be at higher risk than colonization by *Escherichia coli* [13]. A better understanding of this link between colonization and infection is of paramount importance since ESBL-E ICU infections lead to increased healthcare costs, length of stay and mortality [14]. In a non-ICU low-endemy environment, ESBL-E infections among ESBL-E faecal carriers were shown to be a rare event but this remains an issue regarding ICU patients, especially those developing ventilator-associated pneumonia (VAP) [15, 16]. The aim of this work was to assess the ESBL-E dissemination (acquisition and clonal transmission) and subsequent infection among ESBL-E faecal carriers in ICU during a non-outbreak situation.

## Methods

### Design of the study

From January 1^st^ to June 30^th^ 2015, all patients of the medical ICU at Pellegrin Hospital, a 1300-bed tertiary center, were screened by rectal swab at admission and then weekly until they were discharged and isolates were collected. Our ICU is divided into 2 ICU wards of respectively 13 and 14 beds and one 12 beds ward dedicated to post-intensive care, admitting 1200 patients per year with a mean length of stay of 5 days. All patients were hospitalized in single rooms. Standard isolation procedures for all patients and contact isolation procedures for ESBL-E faecal carriers were applied according to the French Society of Hygiene guidelines [17] including hand hygiene procedures, dedicated medical supplies and single room.

For each ESBL-E carriers, demographic information, medical history, exposure to antibiotics for the past 12 months, prior ESBL-E fecal carriage for the past 12 months, hospitalization or health-care facility contact for the past 12 months and mortality at day 28 and year 1 were retrospectively collected through medical records. The patients were characterized as imported carriers if they were colonized at admission (from the community or from a non-ICU medical unit) and acquired carriers if the first screening was negative but any of the weekly screening was subsequently positive. Recording of patients suffering from VAP or pneumonia without intubation was made retrospectively through our prospective patient database. Patient status was assessed by two independent clinicians for all data including the confirmation of VAP diagnosis according to French current guidelines [18]. Bloodstream infection was defined by any positive blood culture except for coagulase-negative *Staphylococcus*.

### Samples processing

Each rectal swab was inoculated on the chromID ESBL® plate for 16-24hours at 37°C. Confirmation of the presence of ESBL was assessed by MAST AMPC&ESBL detection discs D68C® (Mast Group). Identification of each selected colony was assessed by mass spectrometry (Maldi Biotyper Microflex®, Brucker). If 2 different ESBL-E isolates were identified on a screening test, each isolate was characterized individually. In case of positive screening at admission, the isolates collected during weekly screenings were considered as duplicate if being the same species and carrying the same resistance genes and not investigated. Susceptibilities to ceftolozane-tazobactam and ceftazidime-avibactam were assessed by E-test® (BioMérieux). ESBL-E isolates were then stored at -20°C.

### ESBL type determination

Detection and characterization of *bla* genes were performed by multiplex PCRs for *bla*_CTX − M_, *bla*_SHV_, *bla*_TEM_, and *bla*_OXA-1_ and then confirmed by simplex PCRs [19].

### Clonality assessment by pulsed-field gel electrophoresis

The clonality of ESBL-E dissemination in the ICU was determined by pulsed-field gel electrophoresis (PFGE) of *XbaI*-digested genomic DNA of all collected ESBL-E isolates available as previously described [20]. Patterns were visually compared and analyzed according to previously reported criteria [21]. Plasmids were not analyzed by PFGE as concerns exist about its discriminative power.

### Statistics analysis

Quantitative variables were summarized as mean ± standard deviation or median (interquartile range). Categorical data were summarized as count (%).

## Results

### ESBL prevalence in ICU patients

**Fig. 1:**
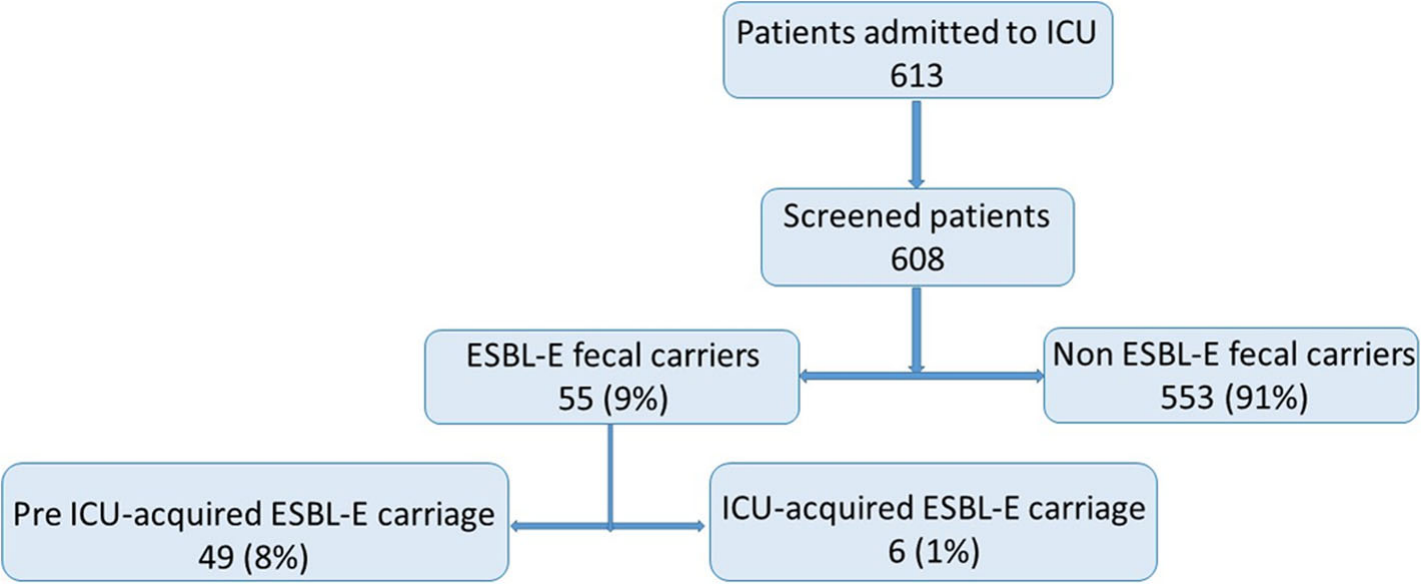
Flow-chart

Among 613 patients admitted to ICU during the 6 months study-period, 608 were screened (371 (61%) only at admission and 237 (39%) at admission and weekly during their ICU stay). Fifty-five (9%) were positive for ESBL-E faecal carriage. No ESBL-E outbreak was detected during the study. Only 6 (1%) patients acquired ESBL-E faecal carriage during their ICU stay (Figure 1): 2 came from the community and 4 were already hospitalized at our universitary hospital before the transfer in ICU. Mean time to acquisition was 31 (±17) days, median time 30 (4-53) days. Each patient who acquired ESBL-E during ICU stay received several courses of broad-spectrum antimicrobial therapy between time of admission in ICU and ESBL-E acquisition.

### ESBL-E faecal carriers’ characteristics and risk factors

ESBL-E faecal carriers are described in Table 1. ESBL-E faecal carriers were mainly colonised with *Escherichia coli* (37/55, 67%), 37/47 (79%) patients had at least 1 risk factor of EBL-E faecal carriage with 32/47 (68%) having several risk factors (Table 2) including previous antimicrobial therapy Table 2.

**Table 1:** ESBL-E faecal carriers’ characteristics at admission

ESBL-E faecal carriers’ characteristics at admission	Patients	(%age)
Age (years, mean ± SD)	61	± 22
Sex ratio (women/men)	0,49	18/37
Simplified Acute Physiology Score 2 (mean ± SD)	50	± 32
Admission from community	24/55	44%
Admission from another hospitalization unit	31/55	56%
D28 mortality	19/54	35%
1-year mortality	20/45	44%
*Escherichia coli* carriage	37/55	67%
*Klebsiella pneumoniae* carriage	16/55	29%
*Citrobacter koseri* carriage	2/55	3%
*Citrobacter freundii* carriage	1/55	3%
*Serratia fonticola* carriage	1/55	1%

**Table 2:** ESBL-E faecal carriage risk factors

ESBL-E faecal carriage risk factors	Patients	(%age)
ESBL-E previous colonization within 12 months	16/55	29%
Travel in a ESBL-E endemic area within 12 months	2/55	3,6%
Previous hospitalization within 12 months	37/55	67%
Health-care associated	26/55	47%
Previous antimicrobial therapy within 12 months	35/47	74%
Penicillin	26/41	63%
3rd generation cephalosporins	17/41	41%
Fluroquinolones	9/41	22%
Trimethoprim/sulfamethoxazole	7/41	17%
Aminoglycosids	9/41	22%
0 risk factor	10/47	21%
1 risk factor	5/47	11%
2 risk factors	9/47	19%
3 risk factors	12/47	26%
4 risk factors	11/47	23%
5 risk factors	0/47 0%	

### ESBL-E clonality assessment by pulsed-field gel electrophoresis (Figs. 2 and 3)

**Fig. 2:**
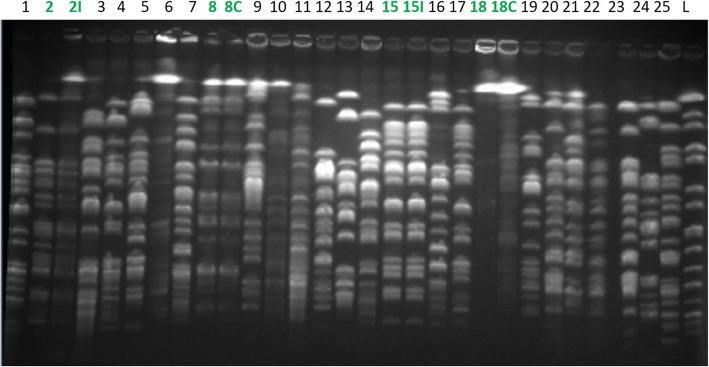
PFGE analysis for *Escherichia.coli* PFGE migration for *Escherichia coli* colonizing isolates. 8C and 18C: duplicate colonizing isolates used as positive control. 2I and 15I: infecting isolates. L: ladder

Fifty-seven ESBL-E isolates from faecal carriage were collected from the 55 patients. Unfortunately, 13 isolates were not available for analysis (loss of ESBL after freezing *n* = 8, the isolate did not grow after – 20°C freezing *n* = 83, and the wrong isolate was frozen at -20°C *n* = 82)). Among these 13 isolates, 10 were imported and 3 acquired in ICU. One of the missing acquired ESBL-E isolate was a *Citrobacter freundii* with no other patient carrying that bacteria during his/her stay in ICU. Forty-four isolates from faecal carriage were available for PFGE and 2 isolates from weekly samplings of imported ESBL-E faecal carriage were considered as duplicate and used as positive control (8C and 18C, Fig. 2).

**Fig. 3:**
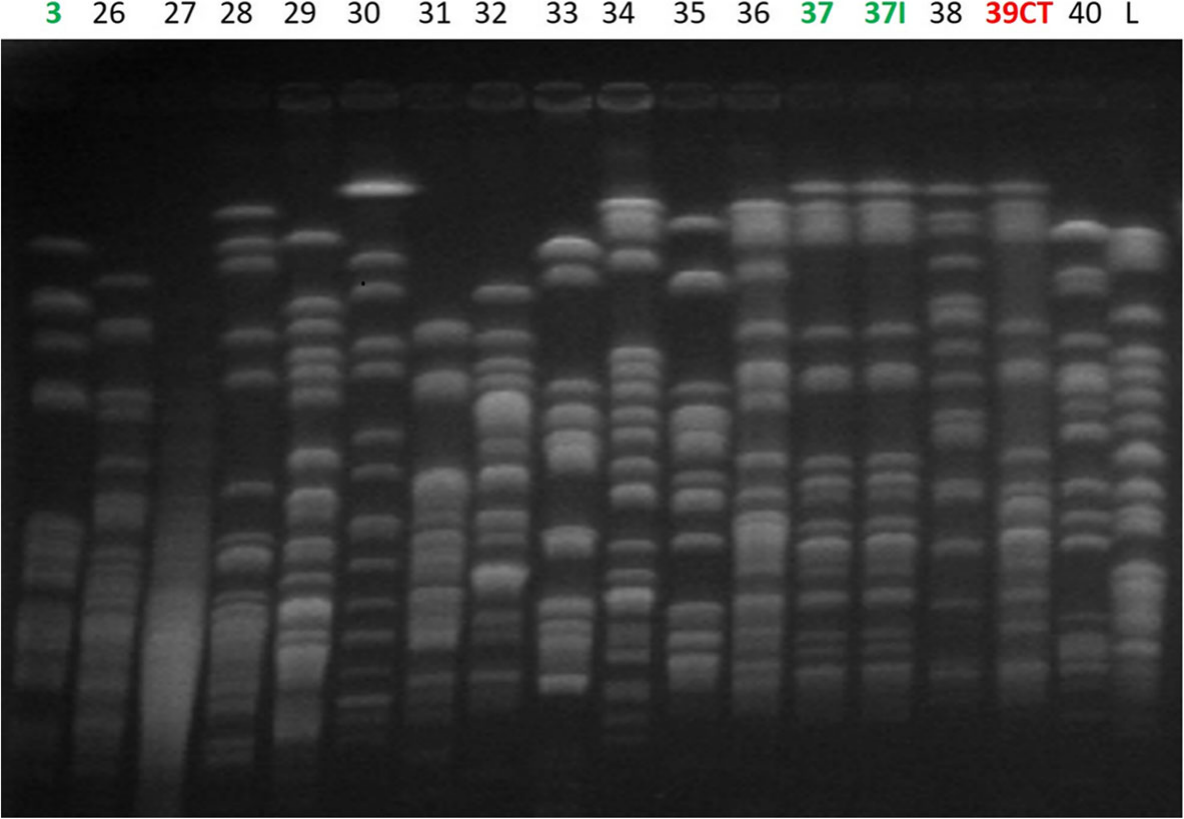
PFGE analysis for *Klebsiella pneumonia* PFGE migration for *Klebsiella pneumoniae* colonizing isolates. Patient 3 was colonized with both *E. coli* and *K. pneumonia*. 37I: infecting strain. 39CT: isolate involved in cross-transmission. L: ladder

No case of cross transmission regarding *E. coli* and only one case of *K. pneumoniae* cross transmission (39CT, Fig. 3) was identified. ESBL gene PCR identified 103 different ESBL-E genes mostly from *bla*_CTX-M_ (43, 42%), *bla*_TEM_ (27, 26%), *bla*_OXA-1_ (18, 17%) and *bla*_SHV_ (14, 15%) groups.

### ESBL-E infections among carriers during ICU stay

Among the 55 ESBL-E faecal carriers, 38 were infected and 16 of them received carbapenems as part of empirical antimicrobial therapy during their ICU stay. Infection sites were distributed as follow: pulmonary infections 23/38, bloodstream infection 9/38 (5 patients having both pulmonary and bloodstream infections), skin infection 5/38, urinary tract infection 4/38, abdominal infection 2/38. Among the 38 infected ESBL-E faecal carriers, a non ESBL-E isolate was identified for 22 patients and 10 patients had no documentation because cultures remain sterile. Thus, only 6/38 patients were subsequently infected by an ESBL-E. None of these 6 ESBL-E infected patients had acquired the ESBL-E faecal carriage during their ICU stay. Respectively for *E. coli* and *K. pneumonia* carriers, 2/37 (6%) and 4/16 (25%) were subsequently infected with ESBL-E.

The 6 ESBL-E infections were as follow: 2 ventilator-associated pneumonias (VAP), 2 pneumonias in non-intubated patients (2 *E. coli* and 2 *K. pneumoniae*), 1 bloodstream infection (*K. pneumoniae*) and 1 urinary tract infection (*K. pneumoniae*). Three isolates involved in pneumonia episodes (1 *K. pneumoniae:* 37I and 2 *E. coli:* 2I and 15I) were also available for PFGE analysis. The PFGE analysis, according to Tenover’s criteria, classified the strains 15I and 37I as indistinguishable from strains 15 and 37 respectively and closely related which means probably the same strain for 2I when compared to strain 2 which confirmed that the same clone was involved in colonisation and infection (Figs. 2 and 3). Both VAP occurred early in the ICU course (2 and 3 days after intubation).

### ESBLE-E VAP prediction among ESBL-E faecal carriers

During the study, 433 patients received mechanical ventilation among whom 39 VAP were observed: 34 non-ESBL-E VAP among the non ESBL-E faecal carriers, 3 non-ESBL-E VAP among the ESBL-E faecal carriers (*n* = 55), 2 ESBL-E VAP among the ESBL-E faecal carriers and 0 ESBL-E VAP among the non ESBL-E faecal carriers. ESBL-E faecal carriage had thus a positive predictive value (PPV) of 40%, a negative predictive value (NPV) of 100%, a sensitivity of 100% and a specificity of 10% for ESBL-E causing the VAP.

### Alternatives to carbapenems

In vitro, all the available isolates (*n* = 44) were susceptible to imipenem, ertapenem, ceftolozane-tazobactam and ceftazidime-avibactam, 43/44 to temocillin and to piperacillin-tazobactam (according to the EUCAST 2016 Version 6.0 breakpoint recommandations).

## Discussion

ESBL-E fecal carriage rate was 9% with only one case of ESBL-E cross-transmission whereas 6 cases of ESBL-E acquisition were observed. In case of subsequent ESBL-E infection, our data demonstrate that the colonizing ESBL-E strain is indeed involved but it remains a rare event conferring poor predictive value to ESBL-E colonization status for subsequent ESBL-E infection.

A prevalence of ESBL-E faecal carriage of 9% is consistent with a previous rate of 13,2% found in another study [22]. Most of the ESBL genes were of CTX-M group as previously described [2, 3]. Only 6 patients (1%) acquired ESBL-E fecal carriage during their stay in ICU and this can be over-estimated. In fact, the rate of false negatives is a major concern regarding ESBL-E screening by rectal swab and 2 patients acquired the ESBL-E in only 4 days, suggesting a possible false negative screening at admission [23].

However, the low rate of ESBL faecal carriage cross transmission (1%), even among patients acquiring faecal carriage in the ICU, underlines the respect of isolation procedures by ICU healthcare givers but also the limitation of these measures as suggested by Tschudin-Sutter et al. [10].

These results are consistent with those of three recent studies but our study is the first assessing ESBL-E clonal dissemination in ICU by PFGE. One shows no case of cross transmission with the respect of the sole standard hygiene precautions in 3 Dutch hospitals but the study did not focus on ICU [11]. The second found only two cases of cross transmission in a ICU with no single room but the major limitation of this work is that no clonal analysis (by PFGE or whole genome sequencing) was performed [22]. The third one found only one case of cross transmission in a medical ICU by repetitive element sequence-based PCR (repPCR) [24]. RepPCRis another tool to assess clonality based on the amplification of repetitive and non-coding parts of the genome but PFGE has a better ability to discriminate between *E. coli* isolates belonging to different subtypes [25]. New techniques such as whole genome sequencing or Maldi-Tof clonality determination will provide an easier and faster assessment of cross-transmission avoiding time- and staff-consuming techniques as PFGE [26, 27].

These data suggest that other mechanisms than cross transmission occur in ICU and should be investigated to better fight ESBL-E faecal carriage acquisition. In fact, besides clonal dissemination, ESBL plasmid-mediated dissemination can occur with horizontal transfer of genetic determinants for antimicrobial resistance which is enhanced during the exposure to antibiotics [28]. Nevertheless, ESBL gene and plasmid incompatibility groups determinations (data not shown for incompatibility groups) do not suggest horizontal transfer to be involved even if definitive conclusions cannot be drawn for 8 of the strains. Another mechanism could correspond to the expansion of a previously present but not detectable ESBL-E clone. This non detectable clone could be acquired in the community or during a previous hospitalization. Antimicrobial therapy before admission or at the early phase of the ICU stay is thought to favor such ESBL-E expansion [29].

The fact that only 6/38 ESBL-E faecal carriers with infection had a proven subsequent ESBL-E infection questions the systematic use of carbapenems in that case, currently a hot topic [30–32]. Piperacillin-tazobactam, ceftazidime-avibactam and ceftolozane-tazobactam could represent safe alternatives to spare carbapenems [33–36]. Regarding piperacillin-tazobactam, the only randomized controlled trial available did not manage to prove the non-inferiority of piperacillin-tazobactam compared with meropenem for the documented treatment of BSI due to 3CG-resistant *E.coli* or *K. pneumoniae* but some limitations apply and so the results should be interpreted cautiously [37, 38]. Nevertheless, in case of high-inoculum or in case of septic shock, carbapenems use remains a gold standard according to the current guidelines [16, 23].

This study confirms that ESBL-E VAP is a rare event even among ESBL-E carriers and that ESBL-E faecal carriage has a very good NPV for ESBL-E VAP but a poor PPV [39] but it demonstrates for the first time that the infecting strain corresponds to the colonizing one in case of subsequent ESBL-E infection. The low rate of VAP (39/433, 9%) in this cohort can decrease the PPV of ESBL-E fecal carriage for subsequent infection.

Besides, infection by ESBL-E seems to be more frequent in the case of *Klebsiella pneumoniae* than *Escherichia coli* as previously suggested [13, 40]. Unfortunately, in front of the small number of events we cannot definitely conclude here.

Our study has some limitations. First some isolates were missing (3 regarding acquired ESBL-E faecal carriage) and we cannot exclude other cases of cross-transmission. Nevertheless, one of the 3 missing isolates was a *Citrobacter freundii* with no other patient carrying a ESBL *Citrobacter freundii* at that time excluding a cross-transmission in that case.

Systematic ESBL-E faecal carriage screening has long been a standard of care for ICU-hospitalized patients but those results question the relevance of screening procedures. In other terms, the paradigm of ESBL-E faecal carriage in ICU patients is changing with a low rate of cross transmission and a majority of imported ESBL-E.

Investigation of the mechanisms leading to plasmid-mediated dissemination or to the expansion of ESBL clones up to detectable ESBL faecal carriage is also needed to resolve ESBL-E colonization issue.

## Conclusions

In this single-centre ICU study, a 9% rate of ESBL-E faecal carriers was observed. Only 1% of patients acquired the ESBL-E faecal carriage during their ICU stay with only one case of cross-transmission. Interestingly, as far as we know, our study demonstrates for the first time by PFGE that the colonizing strain is indeed the infecting one in case of subsequent ESBL-E infection. Nevertheless, only 6/55 ESBL-E faecal carriers were subsequently infected by a proven ESBL-E strain. A negative ESBL-E faecal carriage ruled out its participation in VAP but positive predictive value was poor. The paradigm of ICU transmitted ESBL-E faecal carriage may be changing with a majority of imported ESBL-E and a low rate of cross transmission. Relevance of systematic ESBL-E faecal screening at ICU admission and during ICU stay needs further investigation.

## Data Availability

The datasets generated during the current study are not publicly available due to the recommendations of French law regarding patients’ data but are available from the corresponding author on reasonable request.

## Abbreviations

ESBL-E: Extended-spectrum beta-lactamase producing *Enterobacterales*
ICU: Intensive care unit
NPV: Negative predictive value
PCR: Polymerase-chain reaction
PFGE: Pulsed-field gel electrophoresis
PPV: Positive predictive value
repPCR: Repetitive element sequence-based PCR
VAP: Ventilator-associated pneumonia
